# Disfunção Microvascular Coronariana: Isso Realmente Importa na Doença de Chagas?

**DOI:** 10.36660/abc.20201219

**Published:** 2020-12-01

**Authors:** Maria do Carmo Pereira Nunes

**Affiliations:** 1 Hospital das Clínicas Faculdade de Medicina Universidade Federal de Minas Gerais Belo HorizonteMG Brasil Hospital das Clínicas, Faculdade de Medicina da Universidade Federal de Minas Gerais, Belo Horizonte, MG - Brasil

**Keywords:** Doença de Chagas/fisiopatologia, Microcirculação, Fatores de Risco, Inflamação, Aterosclerose, Isquemia Miocárdica

Há um crescente reconhecimento de que os distúrbios que afetam a estrutura e a função da microcirculação coronariana podem agir como mediadores-chave dos sintomas e prognóstico dos pacientes.^1^ O subconjunto de distúrbios que afetam a microcirculação é denominado disfunção microvascular coronariana (DMC), que é expressa como a incapacidade das artérias coronárias de se dilatarem de forma adequada para atender à demanda de oxigênio do miocárdio e/ou como a redução abrupta no fluxo sanguíneo coronário relacionado ao espasmo coronário.^2^

Nas últimas 2 décadas, a DMC tem sido investigada ativamente em várias condições cardíacas em um amplo espectro de fatores de risco cardiovascular.^2 , 3^ A disfunção microvascular é desencadeada por inflamação sistêmica de baixo grau induzida por fatores de risco cardiovascular convencionais, incluindo hipertensão, diabetes, obesidade, dislipidemia e idade avançada.^1 , 4^ Esse espectro de anormalidades provavelmente pode ser ampliado pela presença de aterosclerose epicárdica.

Apesar de extensas investigações, os mecanismos subjacentes à DMC não são totalmente compreendidos.^1^ Vários mecanismos funcionando isoladamente ou em combinação foram propostos para explicar a patogênese desse distúrbio. Há evidências crescentes demonstrando que as anormalidades funcionais, incluindo disfunção endotelial e das células musculares lisas, têm papel fundamental na regulação do fluxo sanguíneo coronariano em resposta às necessidades cardíacas de oxigênio.^2^ Além disso, a aterosclerose difusa nas artérias coronárias epicárdicas também desempenha um papel ao afetar a função da microvasculatura. De fato, as evidências contemporâneas apoiam a coexistência da DMC com aterosclerose coronariana obstrutiva na maioria dos pacientes afetados.^1^

Embora não haja uma definição universalmente aceita para a DMC,^5^ ela geralmente é definida como a síndrome clínica da angina, evidência de isquemia miocárdica na ausência de doença arterial coronariana obstrutiva.^6^ Como a microcirculação coronária está além da resolução da angiografia coronariana, o diagnóstico de DMC é baseado na avaliação funcional das artérias coronárias, que pode ser realizada por métodos invasivos e não-invasivos. A angiografia coronária invasiva com testes de função da artéria coronária é a abordagem preferencial para avaliar pacientes com DMC. Uma avaliação abrangente da função microvascular inclui o teste dos dois principais mecanismos de disfunção microvascular. A vasodilatação microvascular do endotélio deficiente é medida pela reserva de fluxo coronariano (RFC) e pelo índice de resistência microcirculatória (IRM) e disfunção dependente do endotélio deficiente, que avalia a indução de espasmo epicárdico ou microvascular após uma injeção intracoronária de acetilcolina.^2^ O teste não-invasivo pode avaliar apenas marcadores substitutos da função coronária.

Em contraste com a doença arterial coronariana obstrutiva em que as anormalidades da perfusão têm distribuição regional, o comprometimento miocárdico na DMC pode ser um processo generalizado, resultando em anormalidades difusas da perfusão miocárdica.^2^ A tomografia por emissão de pósitrons (PET) é o padrão de referência para avaliação não-invasiva do fluxo sanguíneo miocárdico.

Na doença de Chagas, estudos experimentais e clínicos sustentam a hipótese de que a DMC pode estar implicada na patogênese do dano miocárdico.^7 - 10^ A infecção por *T. cruzi* pode levar a anormalidades microvasculares funcionais e estruturais, que contribuem para a isquemia miocárdica e sintomas. Semelhante à doença arterial coronariana, a cardiomiopatia chagásica pode afetar o miocárdio de maneira regional com anormalidades segmentares da contratilidade. Entretanto, apesar desses achados sugerirem isquemia miocárdica, a angiografia coronariana invariavelmente demonstra ausência de aterosclerose obstrutiva afetando as artérias coronárias epicárdicas.^8^ Além disso, o comprometimento da vasodilatação coronariana dependente do endotélio em resposta à acetilcolina foi relatado em pacientes com cardiomiopatia chagásica.^11^ Ademais, estudos anteriores mostraram anormalidades de perfusão miocárdica em pacientes com artérias coronárias epicárdicas normais, reforçando o conceito de regulação anormal do fluxo miocárdico em nível microvascular.^8 , 9^

Com essa revisão em mente, é interessante ler o artigo de Campos et al.,^12^ nesta edição dos Arquivos Brasileiros de Cardiologia. Neste estudo, os investigadores avaliaram pacientes encaminhados para angiografia coronária invasiva apresentando angina e suspeita de isquemia miocárdica. Entre os 1.292 pacientes submetidos à angiografia coronária, 247 apresentavam doença arterial coronariana epicárdica não-significativa, 101 pacientes preencheram os critérios de inclusão e foram incluídos no estudo. Posteriormente, esses pacientes com suspeita de DMC foram estratificados em dois grupos de acordo com o diagnóstico de doença de Chagas, sendo 15 pacientes com doença de Chagas e 86 com doença não-Chagas. Os pacientes com doença de Chagas apresentaram maior prevalência de anormalidades regionais da contratilidade miocárdica e menor fração de ejeção do ventrículo esquerdo em comparação com aqueles que tinham suspeita de DMC relacionada aos outros fatores de risco cardiovascular. Este estudo destacou a importância da doença de Chagas como potencial etiologia para DMC, independentemente dos fatores de risco convencionais para esse distúrbio.

No presente estudo, o diagnóstico de DMC foi baseado na ausência de doença coronariana epicárdica obstrutiva. No entanto, a DMC é definida pela presença da reserva de fluxo coronariano limitado e /ou disfunção endotelial coronariana associada à tríade clássica de dor torácica persistente, ausência de doença arterial coronariana obstrutiva e evidência objetiva de isquemia miocárdica induzida por testes de estresse. De fato, a perfusão miocárdica avaliada pela cintilografia (SPECT) foi realizada apenas em um subgrupo de 19 pacientes (18,8%), o que foi uma limitação do estudo. Portanto, mais estudos sobre DMC no contexto da cardiomiopatia chagásica são necessários para avançar nosso entendimento nesta área.
