# Supplementary material for: Disfunção Microvascular Coronariana: Isso Realmente Importa na Doença de Chagas?
Source: Arq Bras Cardiol. 2020 Dec 1;115(6):1102–3. [Article in Portuguese] doi: 10.36660/abc.20201219 (PMC8133722; doi:10.36660/abc.20201219)
Supplement: Translation

# Translation: Coronary Microvascular Dysfunction: Does it Really Matter in Chagas Disease?

**Authors:** Maria do Carmo Pereira Nunes

**Affiliations:** 1 Brazil Hospital das Clínicas, Faculdade de Medicina da Universidade Federal de Minas Gerais, Belo Horizonte, MG - Brazil

**Keywords:** Chagas Diseases/physiopathology, Microcirculation, Risk Factors, Inflammation, Atherosclerosis, Myocardial Ischemia

There is a growing recognition that disorders affecting the structure and function of the coronary microcirculation can act as key mediators of patient symptoms and prognosis.^1^ The subset of disorders affecting the microcirculation is termed coronary microvascular disease (CMD), which is expressed as either the inability of the coronary arteries to dilate appropriately to meet myocardial oxygen demand and/or as the abrupt reduction in coronary blood flow related to coronary spasm.^2^

For the past 2 decades, CMD has been actively investigated in various cardiac conditions across a broad spectrum of cardiovascular risk factors.^2 , 3^ Microvascular dysfunction is triggered by low-grade systemic inflammation induced by conventional cardiovascular risk factors, including hypertension, diabetes, obesity, dyslipidemia, and older age.^1 , 4^ This spectrum of abnormalities may likely be magnified by the presence of epicardial atherosclerosis.

Despite extensive investigations, the mechanisms underlying CMD are not fully understood.^1^ Several mechanisms operating alone or in combination have been proposed to explain the pathogenesis of this disorder. There is increasing evidence demonstrating that functional abnormalities including endothelial and smooth muscle cell dysfunction have a fundamental role in the regulation of coronary blood flow in response to cardiac oxygen requirements.^2^ Additionally, diffuse atherosclerosis in the epicardial coronary arteries also plays a role in affecting the microvasculature function. Indeed, contemporary evidence supports the coexistence of CMD with obstructive coronary atherosclerosis in most affected patients.^1^

Although there is no universally accepted definition for CMD,^5^ it is usually defined as the clinical syndrome of angina, evidence of myocardial ischemia in the absence of obstructive coronary artery disease.^6^ Since coronary microcirculation is beyond the resolution of coronary angiography, the diagnosis of CMD is based on the functional assessment of the coronary arteries, which can be performed using both invasive and non-invasive methods. Invasive coronary angiography with tests of coronary artery function is the preferential approach to evaluate patients with CMD. A comprehensive assessment of microvascular function includes testing the two main mechanisms of microvascular dysfunction. Impaired endothelium microvascular vasodilatation is measured by coronary flow reserve (CFR) and by the index of microcirculatory resistance (IMR), and impaired endothelium-dependent dysfunction, which evaluates the induction of epicardial or microvascular spasm after an intracoronary injection of acetylcholine.^2^ Non-invasive testing can only evaluate surrogate markers of coronary function.

In contrast to obstructive coronary artery disease, in which perfusion abnormalities have regional distribution, myocardial impairment in CMD may be a generalized process resulting in diffuse myocardial perfusion abnormalities.^2^ Positron-emission tomography (PET) is the reference standard for non-invasive assessment of myocardial blood flow.

In the setting of Chagas disease, experimental and clinical studies support the hypothesis that CMD may be implicated in the pathogenesis of myocardial damage.^7 - 10^ . *T. cruzi* infection may lead to functional and structural microvascular abnormalities, which contribute to myocardial ischemia and symptoms. Similar to coronary artery disease, Chagas cardiomyopathy may affect the myocardium in a regional manner, with localized segmental wall motion abnormalities. However, despite these findings suggesting myocardial ischemia, coronary angiography invariably demonstrate the absence of obstructive atherosclerosis affecting the epicardial coronary arteries.^8^ Additionally, impairment of endothelium-dependent coronary vasodilatation in response to acetylcholine has been reported in patients with Chagas cardiomyopathy.^11^ Moreover, previous studies showed myocardial perfusion abnormalities in patients with normal epicardial coronary arteries, supporting the concept of abnormal myocardial flow regulation at the microvascular level.^8 , 9^

With this revision in mind, it is interesting to read the paper from Campos et al.^12^ in this issue of *Arquivos Brasileiros de Cardiologia* . In this study, the investigators evaluated patients referred for invasive coronary angiography presenting with angina and suspected of myocardial ischemia. Of the 1,292 patients undergoing coronary angiography, 247 had non-significant epicardial coronary artery disease, 101 patients met the inclusion criteria and were enrolled in the study. Subsequently, these patients with suspected CMD were stratified into two groups according to the diagnosis of Chagas disease, being 15 patients with Chagas and 86 with non-Chagas disease. The patients with Chagas disease showed a higher prevalence of regional wall motion abnormalities and lower left ventricular ejection fraction, when compared with those who had suspected CMD related to other cardiovascular risk factors. This study highlighted the importance of Chagas disease as a potential etiology for CMD, regardless of conventional risk factors for this disorder.

In the current study, the diagnosis of CMD was based on the absence of obstructive epicardial coronary obstructive disease. However, CMD is defined by the presence of limited coronary flow reserve and/or coronary endothelial dysfunction associated with the classic triad of persistent chest pain, absence of obstructive coronary artery disease and objective evidence of myocardial ischemia induced by stress tests. Indeed, myocardial perfusion assessed with SPECT scintigraphy was performed only in a subset of 19 patients (18.8%), which was a limitation of the study. Therefore, further studies on CMD in the context of Chagas cardiomyopathy are needed to advance our understanding on this field.
